# Structural stigma and sexual minority men’s depression and suicidality: A multi-level examination of mechanisms and mobility across 48 countries

**DOI:** 10.1093/eurpub/ckac129.195

**Published:** 2022-10-25

**Authors:** J Pachankis, ML Hatzenbuehler, R Bränström, AJ Schmidt, RC Berg, K Jonas, M Pitoňák, S Baros, P Weatherburn

**Affiliations:** 1 Department of Social and Behavioral Sciences, Yale School of Public Health, New Haven, USA; 2 Department of Psychology, Harvard University, Boston, USA; 3 Department of Clinical Neuroscience, Karolinska Institutet, Stockholm, Sweden; 4 Department of Public Health, Environments and Society, LSHTM, London, UK; 5 Department of Community Medicine, University of Tromso, Tromso, Norway; 6 Faculty of Psychology and Neuroscience, Maastricht University, Maastricht, Netherlands; 7 Centre of Epidemiological and Clinical Research, National Institute of Mental Health, Klecany, Czechia; 8 Institute of Public Health of Serbia, Centre for Diseases Prevention and Control, Belgrade, Serbia

## Abstract

**Background:**

Sexual minority men are at greater risk of depression and suicidality than heterosexuals. Stigma, the most frequently hypothesized risk factor for this disparity, operates across socioecological levels-structural (e.g., laws), interpersonal (e.g., discrimination), and individual (e.g., self-stigma). However, there is limited data on whether changes in structural stigma, such as when a stigmatized person moves to a lower stigma context, affect mental health, and on the mechanisms underlying this association

**Methods:**

The current study uses data from the 2017/18 European Men-who-have-sex-with-men Internet Survey (n = 123,428), which assessed mental health and psychosocial mediators. We linked these data to an objective indicator of structural stigma related to sexual orientation in respondents’ countries of origin (N = 178) and receiving (N = 48) countries

**Results:**

Among respondents who moved from higher-to-lower structural stigma countries (n = 11,831), longer exposure to the lower structural stigma environments of their receiving countries was associated with a significantly: 1) lower risk of depression and suicidality; 2) lower odds of concealment, internalized homonegativity, and social isolation; and 3) smaller indirect effect of structural stigma on mental health through these mediators.

**Conclusions:**

This study provides evidence that structural stigma is associated with the mental health of sexual minority men, both through proximal experiences and as a function of length of exposure to structurally diverse contexts, at least for those who move higher-to-lower structural stigma contexts. Findings suggest the importance of routinely assessing life-course structural influences on mental health and deploying interventions to address those influences.

